# Highlight report: Occupational urinary bladder cancer

**DOI:** 10.17179/excli2017-1037

**Published:** 2017-12-19

**Authors:** H.M. Bolt

**Affiliations:** 1 IfADo, Leibniz Research Centre for Working Environment and Human Factors, Dortmund

## ⁯

Recently, Cordula Lukas and colleagues from TU Dortmund have revisited the relationship between the occurrence of urinary bladder cancer and polymorphisms of xenobiotic metabolizing enzymes (Lukas et al., 2017[10]). Currently, the fraction of occupationally related bladder cancer is estimated as 7.1 % in men and 1.9 % in women (Rushton et al., 2012[13]). 

The majority of these occupational bladder carcinomas are associated with exposure to aromatic amines and azo dyes. Lukas et al. (2017[10]) analyzed polymorphisms of N-acetyltransferase 2 (NAT2), glutathione S-transferase M1 (GSTM1), glutathione S-transferase T1, UDP-glucuronosyltransferase 1A (UGT1A), a polymorphism close to the oncogene c-myc (rs9642880) and a polymorphism close the p53 family member TP63 (Lukas et al., 2017[10]). The strongest association with occupational urinary bladder cancer was obtained for GSTM1 and UGT1A, especially when both are co-occurring. The GSTM1 deletion was observed more frequently in varnishers and painters (Lukas et al., 2017[10]). This was associated with exposure to aromatic amines and carbolineum. Interestingly, the polymorphisms were not only associated with increased bladder cancer risk but also with shorter relapse-free times (Lukas et al., 2017[10]). It remains to be analyzed why GSTM1 and UGT1A influence bladder cancer prognosis.

Associations with polymorphisms have been studied in more than 1800 diseases and thousands of SNP associations have been found but typically SNPs only explain a minor share of the variance (Liaqat et al., 2015[9]; Hashemi et al., 2015[4]; Saadat, 2016[14]; Malik et al., 2015[11]). The first genome-wide association study in urinary bladder cancer has been studied approximately ten years ago (Kiemeney et al., 2008[6]). Meanwhile, further studies have identified and validated fifteen genomic regions that are associated with increased risk of bladder cancer and studied the relevance of exposure to carcinogens (Figueroa et al., 2016[2]; Rothman et al., 2010[12]; Selinski et al., 2017[16][18], 2016[17], 2015[19]); Höhne et al., 2017[5]; Ebbinghaus et al., 2017[1]; Krech et al., 2017[7], 2016[8]; Gundert-Remy et al., 2015[3]). It is of particular relevance that several polymorphisms may statistically interact to cause a higher risk than each of the individual variants (Selinski, 2017[15]). While variants of GSTM1 and UGT1A were most relevant in occupational bladder cancer this is not observed for NAT2. In future, studies are required that help to understand the discrepancy why NAT2 was relevant in cohorts enrolled in the past but not or much less in more recently collected case-control series.
